# Seed-induced Aβ deposition alters neuronal function and impairs olfaction in a mouse model of Alzheimer’s disease

**DOI:** 10.1038/s41380-022-01686-5

**Published:** 2022-07-22

**Authors:** Stephanie Ziegler-Waldkirch, Marina Friesen, Desirée Loreth, Jonas-Frederic Sauer, Solveig Kemna, Alexandra Hilse, Daniel Erny, Christina Helm, Paolo d´Errico, Marco Prinz, Marlene Bartos, Melanie Meyer-Luehmann

**Affiliations:** 1 Department of Neurology, Medical Center – University of Freiburg, 79106 Freiburg, Germany; 2 Faculty of Medicine, University of Freiburg, 79110 Freiburg, Germany; 3 Faculty of Biology, University of Freiburg, 79110 Freiburg, Germany; 4 Institute of Cellular and Integrative Physiology, University Medical Center Hamburg-Eppendorf, 20246 Hamburg, Germany; 5 Institute for Physiology I, Systemic and Cellular Neurophysiology, University of Freiburg, 79104 Freiburg, Germany; 6 Institute of Neuropathology, University of Freiburg, 79106 Freiburg, Germany; 7 Berta-Ottenstein-Programme, Faculty of Medicine, University of Freiburg, 79110 Freiburg, Germany; 8 Center for Basics in NeuroModulation (NeuroModulBasics), Faculty of Medicine University of Freiburg, 79110 Freiburg, Germany; 9 Signalling Research Centres BIOSS and CIBSS, University of Freiburg, 79104 Freiburg, Germany

**Keywords:** Neuroscience, Stem cells, Cell biology

## Abstract

Alzheimer’s disease (AD) is characterized by the accumulation of amyloid-β (Aβ) which ultimately forms plaques. These Aβ deposits can be induced in APP transgenic mouse models by prion-like seeding. It has been widely accepted that anosmia and hyposmia occur during the early stages of AD, even before cognitive deficits are present. In order to determine the impact of seed-induced Aβ deposits on olfaction, we performed intracerebral injections of seed-competent brain homogenate into the olfactory bulb of young pre-depositing APP transgenic mice. Remarkably, we observed a dramatic olfactory impairment in those mice. Furthermore, the number of newborn neurons as well as the activity of cells in the mitral cell layer was decreased. Notably, exposure to an enriched environment reduced Aβ seeding, vivified neurogenesis and most importantly reversed olfactory deficits. Based on our findings, we conclude that altered neuronal function as a result of induced Aβ pathology might contribute to olfactory dysfunction in AD.

## Introduction

Alzheimer’s disease (AD) is a neurodegenerative disease that is characterized by cognitive decline and memory impairment. In addition, olfactory dysfunction is an early symptom that has been proposed as a possible biomarker to assess the onset and progression of AD [1]. Studies of olfaction in AD have shown a number of deficits such as impaired odor identification, detection, recognition, sensitivity and discrimination [2, 3]. It is also well established that patients with AD exhibit olfactory deficits and impaired odor identification much earlier than during normal aging or the onset of dementia [4–6]. Yet, the mechanism behind this dysfunction remains poorly understood. In line with these functional impairments, the two neuropathological hallmarks of AD, senile plaques and neurofibrillary tangles, are encountered in the olfactory bulb of AD patients and in mouse models of AD, suggesting that it is one of the first sites undergoing pathological changes [1, 7–12]. Interestingly, in AD patients Aβ depositions can be found especially in the olfactory glomerular layer [13] and the anterior olfactory nucleus [14].

In general, the aggregation of Aβ is considered an essential early trigger in AD pathogenesis that leads to neurofibrillary tangles, neuronal dysfunction and dementia [15, 16]. Ample evidence from in vivo seeding model studies in the hippocampus suggests that Aβ aggregation can be initiated by prion-like seeding [17–21]. Interestingly, such seed-induced Aβ deposits were recently shown to impair memory and to diminish adult neurogenesis [22].

Importantly, the olfactory bulb is constantly supplied with newly generated neurons from the subventricular zone (SVZ) of the lateral ventricles which is one of the two neurogenic niches in the adult mammalian brain [23–26]. From the SVZ, the progenitor cells migrate through the rostral migratory stream (RMS) to the olfactory bulb, where neuroblasts mature into olfactory bulb interneurons. Those newborn interneurons are required for odor detection, discrimination, olfactory memory and responses (e.g., avoidance) [27–29]. Newborn granule and periglomerular cells are continuously added to the olfactory system to modulate the activity of mitral and tufted cells, which in turn project to other brain areas including the piriform cortex, entorhinal cortex and amygdala [30–33].

Despite compelling evidence that seed-induced Aβ deposits in the hippocampus elicit neuronal functional deficits and behavioral phenotypes [22], it remains unknown whether seed-induced Aβ deposits in the olfactory bulb have an impact on the cellular level and on olfaction. Addressing these questions is however fundamental to advance our understanding of the very early disease stages and of disease progression.

## Methods

### Animals

We used heterozygous 5xFAD transgenic mice coexpressing human APP^K670N/M671L (Sw)+I716V (Fl)+V717I(Lo)^ and PS1^M146L+L286V^ under the control of the neuron-specific Thy-1 promoter [34] and heterozygous APP23 transgenic mice expressing human APP^K670N/M671L^ [35]. We backcrossed heterozygous 5xFAD and APP23 mice to C57BL/6 mice to generate heterozygous 5xFAD, APP23 and non-transgenic littermates. For the present study only male mice were used to minimize variability and reduce sample size. Animals were group-housed under specific pathogen-free conditions. Mice were kept under a 12 h light, 12 h dark cycle with food and water *ad libitum*. All animal experiments were carried out in accordance with the policies of the state of Baden-Württemberg under license number G13-093, G18-136 and G20-047.

### Preparation of brain homogenates for intracerebral injections

Mouse brain homogenates were derived from 12-month-old plaque bearing heterozygous 5xFAD transgenic mice and aged matched non-transgenic littermates or from a 21-month-old APP23 transgenic mouse. Homogenates were obtained from the whole mouse brain. Brain tissue samples were fresh frozen and stored at −80 °C until use. Samples were homogenized in sterile phosphate-buffered saline (PBS) at 10% (w/v) and sonicated 3 × 5 s (30% amplitude, Digital Sonifier W-250D, Branson Ultrasonics). The crude brain homogenate was centrifuged for 5 min (at 3000 × g, 4 °C) and the supernatant was stored at −80 °C until use.

### Intracerebral stereotactic injections

Mice were anesthetized via intraperitoneal injection of a mixture of ketamine (100 mg/kg body weight) and xylazine (5 mg/kg body weight) in saline. For bilateral stereotactic injections of brain homogenates, a Hamilton syringe was placed into the olfactory bulb (AP + 5.0 mm; L +/−1.0 mm; DV −1.0 mm) of 8 weeks old male 5xFAD mice, 6-month-old APP23 mice or WT. Mice were either injected with 5xFAD (or APP23) transgenic brain homogenate or WT brain homogenate (3 µl per hemisphere at an injection speed of 1.25 µl/min) or were left uninjected as controls. After each injection, the needle was kept in place for additional 2 minutes before it was slowly withdrawn. The surgical site was cleaned with sterile saline and the incision sutured. Mice were sacrificed either 4, 8, 12, or 16 weeks (5xFAD) or 4 and 6 months (APP23) p.i.

### BrdU administration

For detection of slow dividing cells in the adult brain, 5-bromo-2-deoxyuridine (BrdU; Sigma, B5002) was given via drinking water (1 mg/ml) at 9 weeks post-injection for 2 weeks as described before [22, 36, 37]. BrdU solution was prepared in sterile water, protected from light exposure and changed once a week. Then BrdU was replaced with normal water for 2 more weeks. Mice were perfused two weeks after BrdU treatment was stopped.

### Histology

Mice were transcardially perfused with 20 ml of ice-cold PBS followed by 20 ml of ice-cold 4% paraformaldehyde in PBS. Brains were isolated and postfixed in 4% PFA (Roti®-Histofix, Roth) for 24 h, followed by incubation in 30% sucrose (in PBS, pH 7.5) for a further 48 h. Frozen brains were cut into 25 µm thick coronal sections on a sliding microtome (SM2000R, Leica Biosystems, Wetzlar, Germany) and collected in 15% Glycerol. Immunohistochemistry was performed using the following antibodies diluted in PBS containing 5% normal goat serum and 0.5% Triton X-100: anti-Aβ (mouse, 1:3000, Covance, 6E10), anti-doublecortin (rabbit, DCX; 1:5000, abcam, ab18723), anti-NeuN (mouse, Merck, 1:200, MAB377), anti-BrdU (rat, abcam, 1:200, ab6326), anti-C-Fos (Merck, 1:1000, ABE457), anti-Iba1 (rabbit, 1:3000 WAKO), anti-CD68 (rat, 1:500, BioRad), anti-Sox2 (goat, 1:250, SC17320), anti-Calretinin (mouse, 1:1000, BD 610908) and anti-Ki67 (rabbit, 1:500, abcam ab15580). Appropriate secondary antibodies conjugated to Alexa 488 or 555 (1:1500) were used. For BrdU staining sections were pre-treated with 2 N HCl at 37 °C for 30 min before starting the staining protocol. NeuroTrace 500/525 (green fluorescent Nissl stain, Thermo Fisher) staining was done according to manufactural instructions.

Dense-core plaques were stained with Thiazine red (Sigma Aldrich, S570435). Staining was done according to standard protocols. In brief, sections were washed 3 times in 1xPBS and incubated in Thiazine red (0.01% solution in 1xPBS) for 5 min at RT followed by 3 × 10 min washes in 1xPBS.

Sections were counterstained with DAPI (Sigma, D9542, 1:10000) and mounted with fluorescence mounting medium (DAKO, S3023).

### Assessment of Aβ and cell analysis

Fluorescence images of brain slices were taken using a Zeiss fluorescent microscope (Axio Imager M2M). For analysis every tenth brain section of a single hemisphere was immunostained. Areas such as the olfactory bulb, mitral cell layer, piriform cortex and the SVZ were defined based on the mouse brain atlas [38]. Total Aβ load was determined by calculating the % areal fraction occupied by Aβ positive staining in the olfactory bulb using the imaging software ImageJ (National Institutes of Health freeware). 5–6 animals per group and 6 sections per animal were analyzed. The sections represented always the same layers in each animal, starting from Bregma 5.0 to Bregma 3.7.

Cell number was quantified by counting the number of positive labeled cells in the area of interest of the animals. 5–7 animals per group and 3–4 sections per animal were analyzed. The 25 µm thick serial coronal sections represented always the same layers in each animal, starting from Bregma 5.0 to Bregma 3.7. The sections for the piriform cortex were chosen between Bregma 2.3 and 1.6.

Cell counting was done in the olfactory bulb and the area of the olfactory bulb was measured with the ImageJ software. Cell counts were performed within a defined volume based on the region of interest and the thickness of the section (25 µm). All analyses were conducted in a blinded manner.

### Environmental enrichment

8 weeks after intracerebral injections into the olfactory bulb transgenic mice and their non-transgenic siblings were housed in an enriched environment (EE) or in standard conditions (SH) for 4 weeks. Mice were housed in groups of 4 mice. The enriched environment consisted of larger cages (40 × 60 cm) that contained 1 running wheel, tunnel systems, small plastic houses and extra nesting material. The animals had free access to the running wheel. At the end of the experiment at the age of 20 weeks all mice were sacrificed.

### Electrophysiology

Mice were deeply anesthetized with an i.p. injection of ketamine/xylazine mixture (100 mg/10 mg per kg body weight) and placed in a stereotaxic frame. A craniotomy was performed over the olfactory bulb, and a custom-made head plate was cemented to the skull. A 64-channel 4-shank silicon probe (Cambridge Neurotech) was inserted into the brain until the mitral cell layer was detected by prominent multi-unit activity. Continuous recordings were sampled at 30 kHz with an openEphys acquisition system. For each shank with an electrode in the mitral cell layer power spectral density was obtained offline by fast-Fourier transform using Welch’s method in 1-s windows.

### Olfaction test

For all olfaction tests mice were used 12 weeks after intracerebral injections into the olfactory bulb. All experiments were done in the morning.

### Buried food test

The buried food (cookie) test was based on the time it took the mice to find a hidden buried cookie in the bedding, as described previously [39]. In brief, mice were exposed to the cookie two days before the test. The next day, mice were fasted 12 h before the test and habituated to the testing room for 1 h. Then mice were placed into a clean cage (41 cm length × 26 cm width × 18 cm height). The test began by placing the mouse in a clean cage containing 3 cm deep bedding. Following 10 min of habituation, a cookie was placed 0.5 cm below the bedding. The timer was started and the latency to find the cookie was recorded. The mouse was considered to have uncovered the cookie when it started to eat the cookie. If the mouse did not find the cookie within 15 min, the trial was ended and the mouse was excluded from the experiment.

The following animal numbers were used: for the two WT groups *n* = 11, 5xFAD uninjected *n* = 13, 5xFAD + WT *n* = 15 and 5xFAD + 5xFAD *n* = 15.

### Habituation/Dishabituation test

The capability of mice to detect and differentiate various odors (non-social odors) was examined with the olfactory habituation/dishabituation test. The test was done according to established protocols, with minor changes [39]. The main aim of this test is to measure an animal’s tendency to investigate novel smells. This phenomenon can be assessed through presenting the mice with a sequence of different odors. Habituation is defined by a decrease in time spent sniffing the same odor. Dishabituation is represented by a reinstatement of olfactory investigation when a novel odor is presented. Prior to testing, mice were allowed to acclimate for 30 min to the test room and a clean test cage with new bedding. Non-social odors (Carl Roth) were prepared on the same day of the test, which included: (1) distilled water; (2) solution with orange extract; (3) solution with cinnamon extract, (4) solution with coconut and (5) solution with pine extract. The solutions were prepared by adding 10 μl of the test extract to 990 μl of mineral oil (Sigma Aldrich, 1:1000 dilution). Stimuli were presented in the following order: water × 3, orange × 3, water, cinnamon × 3, water, coconut × 3, water and pine × 3. A trial period of 60 s was given for each stimulus presented, and thus the time spent sniffing the tip for each stimulus was recorded in seconds using a silent stopwatch. The odors were presented on a piece of whatman paper (1 × 1 cm). There was a 60 s break between each stimulus.

The following animal numbers were used: for the two WT groups *n* = 10, 5xFAD uninjected *n* = 12, 5xFAD + WT *n* = 15 and 5xFAD + 5xFAD *n* = 17.

### Olfactory avoidance test

An olfactory avoidance test was performed as described previously [40]. The mice were habituated to the testing room for 1 h. Then mice were placed into a clean cage (33 cm length × 20 cm width × 12.5 cm height). The test started by placing the mouse in a clean cage containing 3 cm deep bedding. The test cage was divided into two equal areas. Following 10 minutes of habituation to the cage, a cotton swab scented with nTMT (2,4,5-Trimethylthiazole, Sigma Aldrich, 1:100 diluted in water) was placed in one half of the test cage. Avoidance time was measured during a 60 s test time. “Avoidance time” was defined as the time spent in the area without a cotton swab scented with nTMT. Avoidance behavior was represented by an avoidance index as follows: avoidance index = (*P*–50)/50, where *P* is the percentage of avoidance time during a 60 s test period.

The following animal numbers were used: WT *n* = 8, WT + 5xFAD and 5xFAD + WT *n* = 7, 5xFAD uninjected *n* = 9 and 5xFAD + 5xFAD *n* = 10.

### Immunoblot analysis of the olfactory bulb

Mouse olfactory bulb tissue was dissected on ice and homogenized in 10x volume RIPA buffer. After passing the sample 10 times through a syringe needle and incubation at 4 °C for 30 min, the samples were centrifuged at 9000 rpm for 10 min at 4 °C. The supernatant was stored at −20 °C until use. Protein concentration was determined by Pierce BCA Protein Assay Kit (Thermo Fisher Scientific).

Brain homogenates from the olfactory bulb were subjected to SDS-PAGE using 10% SDS or 4–12% NuPAGE Bis-Tris gels (using NuPAGE 4xLDS sample buffer, NuPAGE 10x sample reducing agent and NuPAGE MES SDS running buffer (Invitrogen)).

Proteins were transferred onto a nitrocellulose or PVDF membrane (0.2 µm pore size; Protran, Whatman) and immunoblotted with antibodies specific to BDNF (rabbit, 1:2000, Santa Cruz, sc-456), CCL2 (rabbit, 1:1000, Novus Biologicals NBP2-41209), DCX (rabbit, 1:3000, abcam, ab18723),TNFα (goat, 1:1000, Invitrogen, PA5-46945), β-actin-HRP (mouse, 1:5000, abcam, ab20272), α-tubulin (chicken, 1:1000, abcam, ab89984), anti-chicken IgG HRP-linked Antibody (1:5000, Santa Cruz, sc-2497), anti-goat IgG HRP-linked Antibody (1:3000, Santa Cruz, sc-2033), and anti-rabbit IgG HRP-linked Antibody (1:5000, abcam, ab16284). Proteins were visualized using Clarity Western ECL Substrate (Biorad) or Supersignal West Femto Maximum Sensitivity Substrate (Thermo Fisher Scientific) and ChemiDoc MP Imaging System (Biorad).

Protein levels were analyzed with the ImageLab software and levels of DCX were normalized to the levels of α-Tubulin (DCX % normalized to α-Tubulin). *N* = 4 animals per group were analyzed.

#### Gene expression analysis

Olfactory bulbs were homogenized in extraction buffer (Pico Pure Kit, Life Technologies). Afterwards RNA was isolated with the Arcturus Pico Pure RNA Isolation Kit (Life Technologies) according to the manufacturer’s protocol. Reverse transcription and real-time PCR analysis were performed using the high capacity RNA-to-cDNA-Kit and Gene Expression Master Mix reagents (Applied Biosystems) according to the manufacturer’s recommendations. qPCRs were analyzed with a LightCycler 480 (Roche). For gene expression analysis, we used the following TaqMan Gene Expression Assays: Actb (Mm01205647_g1), Ccl2 (Mm00441242_m1) and Tnfα (Mm00443258_m1).

### Statistical analysis

GraphPad Prism 6 (GraphPad Software, Inc) and the *stats* package of SciPy (www.scipy.org) running under Python 2.7 were used for statistical analysis. All data sets were tested for normality with the D’Agostino-Pearson omnibus K2 normality test or the Shapiro-Wilk test with a significance level set to *p* = 0.05 before the appropriate parametric or nonparametric statistical comparison test was carried out. Student´s *t*-test or Mann-Whitney test or one-way ANOVA or Kruskal-Wallis test followed by Dunn´s posthoc test or Tukey´s multiple comparison test was applied. For correlation the Pearson correlation was used. Reported values are means ± SEM. Significance level α was set to 0.05. *p < 0.05; **p < 0.01; ***p < 0.001.

## Results

### Aβ seeding in the olfactory bulb of 5xFAD mice is mainly located in the subependymal layer and the anterior commissure

**Fig. 1:**
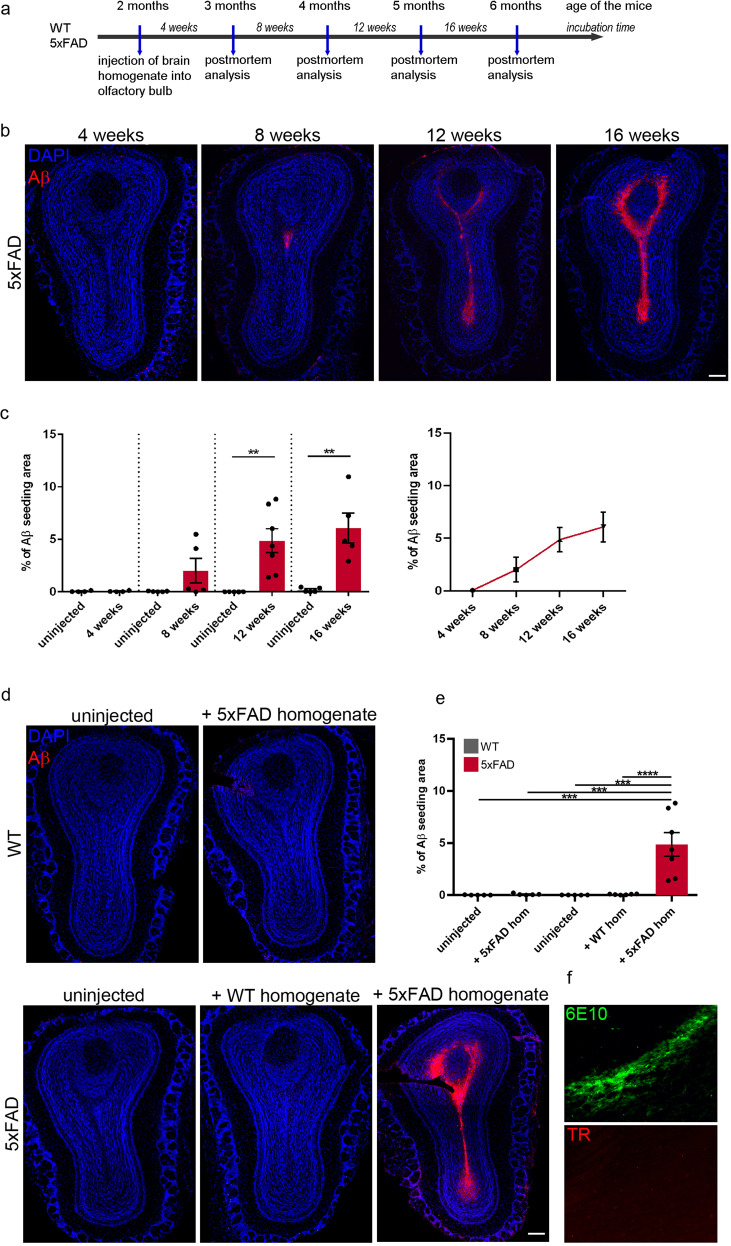
Seed-induced Aβ deposits are located in the subependymal layer and the anterior commissure of the olfactory bulb in 5xFAD mice. **a** Scheme of Aβ seeding experiments in the olfactory bulb of pre-depositing WT and 5xFAD mice. **b** Fluorescence microscopy of Aβ seeding (6E10, red and DAPI, blue). Shown are representative images of olfactory bulbs from male 5xFAD mice sacrificed 4, 8, 12 or 16 weeks p.i. Scale bar represents 200 μm. **c** Quantification of % of Aβ load in the olfactory bulb of 5xFAD mice uninjected or injected with 5xFAD homogenate. Aβ seeding started 8 weeks p.i. and increased with longer incubation times. Each symbol represents data from one mouse (4 weeks: *n* = 4, 8 weeks *n* = 5, 12 weeks *n* = 5/7, 16 weeks *n* = 5). Data are presented as mean ± s.e.m. Significant differences were determined by the Mann–Whitney test (*p* = 0.0053; *p* = 0.0031). **d** Shown are representative immunofluorescence images (6E10, red and DAPI, blue) of olfactory bulbs from WT or 5xFAD mice, uninjected, injected with WT or 5xFAD homogenate, sacrificed 12 weeks p.i. Scale bar represents 200 μm. **e** Quantification of % of Aβ load in the olfactory bulb 12 weeks p.i. 5xFAD mice injected with 5xFAD homogenate have a significantly higher % Aβ load. Data are presented as mean ± s.e.m. Significant differences were determined by the one‐way ANOVA, followed by Tukey’s multiple comparison test F(4, 23) = 12.87, *p* = 0.0002, *p* = 0.0002, *p* = 0.0002, *p* < 0.0001. WT uninjected/+5xFAD and 5xFAD uninjected *n* = 5, 5xFAD + wt *n* = 6, 5xFAD + 5xFAD *n* = 7. **f** Representative images of immunofluorescent staining of Aβ seeding pattern in olfactory bulb from 5xFAD mice injected with 5xFAD brain homogenate with 6E10 (green, upper panel) and Thiazine red (red, lower panel). Scale bar represents 50 µm.

As exogenous induction of Aβ deposition is a time-dependent process [20, 22, 41] we performed intracerebral injections of Aβ containing brain homogenate into the olfactory bulb of 2-month-old pre-depositing 5xFAD mice and analyzed the mice at different time points (4, 8, 12 and 16 weeks post-injection) (Fig. 1a). First signs of seed-induced Aβ deposits appeared 8 weeks post-injection (p.i.), increasing with time at 12 and 16 weeks p.i. (Fig. 1b, c). For our further experiments we decided to use 12 weeks p.i. because uninjected control 5xFAD mice at this age (5 months) are still devoid of any endogenous Aβ plaques and rather start developing Aβ plaques later at the age of 6 months in the granular cell layer of the olfactory bulb (Supplementary Fig. 1). Only 5xFAD mice injected with Aβ-containing brain homogenate developed numerous seed-induced Aβ plaques in the subependymal layer and the anterior commissure (aco), the central region of the olfactory bulb, while no seeded Aβ deposits were found in all other groups tested (WT or control injected animals) (Fig. 1d, e). The seeding pattern is mostly diffuse and negative for Thiazine red (Fig. 1f). In order to generalize these findings, we confirmed our results in APP23 transgenic mice [35] by performing injections into the olfactory bulb of 6-month-old APP23 mice and incubating them either for 4 or 6 months. In line with our previous results, massive seed-induced Aβ depositions were found in the subependymal layer of APP23 mice very similar to seeded 5xFAD mice (Supplementary Fig. 2a, b) suggesting that Aβ seeding in the olfactory bulb is a general phenomenon and not restricted to a specific APP-transgenic mouse model.

### Aβ seeding induces olfactory deficits in young 5xFAD mice

**Fig. 2:**
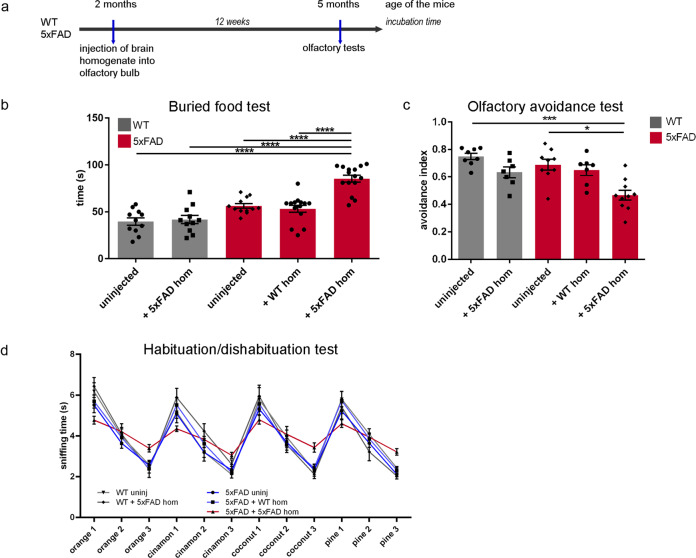
Seed-induced Aβ deposits result in olfactory deficits. **a** Scheme of Aβ seeding experiments in combination with olfactory tests of WT and 5xFAD mice. **b** Quantification of the buried food test. Shown is the time the mice needed to find a hidden cookie. Data are presented as mean ± s.e.m. Significant differences were determined by the one‐way ANOVA, followed by Tukey’s multiple comparison test F(4,59) = 26, *p* < 0.0001. WT uninjected and WT + 5xFAD *n* = 11, 5xFAD uninjected *n* = 13, 5xFAD + WT *n* = 15 and 5xFAD + 5xFAD *n* = 15. **c** Quantification of the olfactory avoidance test. Data are presented as mean ± s.e.m. Significant differences were determined by the one‐way ANOVA, followed by Tukey’s multiple comparison test F(4, 36) = 9.561, *p* = 0.0003; *p* = 0.0145. WT uninjected *n* = 8, WT + 5xFAD and 5xFAD + WT *n* = 7, 5xFAD uninjected *n* = 9 and 5xFAD + 5xFAD *n* = 10. **d** Quantification of the habituation/dishabituation test. Shown is the time the mice explored the scents. Data are presented as mean ± s.e.m. Significant differences were determined by the two‐way ANOVA (*p* < 0.0001). WT uninjected and WT + 5xFAD *n* = 10, 5xFAD uninjected *n* = 12, 5xFAD + WT *n* = 15 and 5xFAD + 5xFAD *n* = 17.

Inspired by our previous study where we reported memory deficits due to Aβ seeding in the hippocampus [22], we proceeded to analyze olfactory functionality and performed olfactory tests in order to directly determine the effect of seed-induced Aβ deposits on olfactory performance. Therefore, we injected 2-month-old 5xFAD mice with Aβ-containing brain homogenate followed by different olfaction tests after an incubation time of 12 weeks (Fig. 2a). Remarkably, seeded 5xFAD mice required significantly more time to find the buried food when compared to all other groups (Fig. 2b). Moreover, during olfactory avoidance tests, seeded 5xFAD mice spent significantly more time in the avoidance area (Fig. 2c), again indicative of olfactory deficits. 5xFAD mice injected with WT homogenate did not show any differences in olfactory behavior compared to the other control groups, indicating that the injection itself has no effect on olfaction. Since it has been reported that AD patients also show difficulties in discriminating different odors [42, 43], we finally conducted a habituation/dishabituation test. Indeed, 5xFAD mice injected with Aβ-containing brain homogenate had profound problems differentiating between familiar and unfamiliar odors (Fig. 2d), while all control groups were able to discriminate between different odors, corroborating the results obtained with the buried food and avoidance tests. Thus, Aβ seeding seems to exert a direct influence on the olfactory behavior of mice.

### Aβ seeding alters adult neurogenesis and neuronal activity in the olfactory bulb

**Fig. 3:**
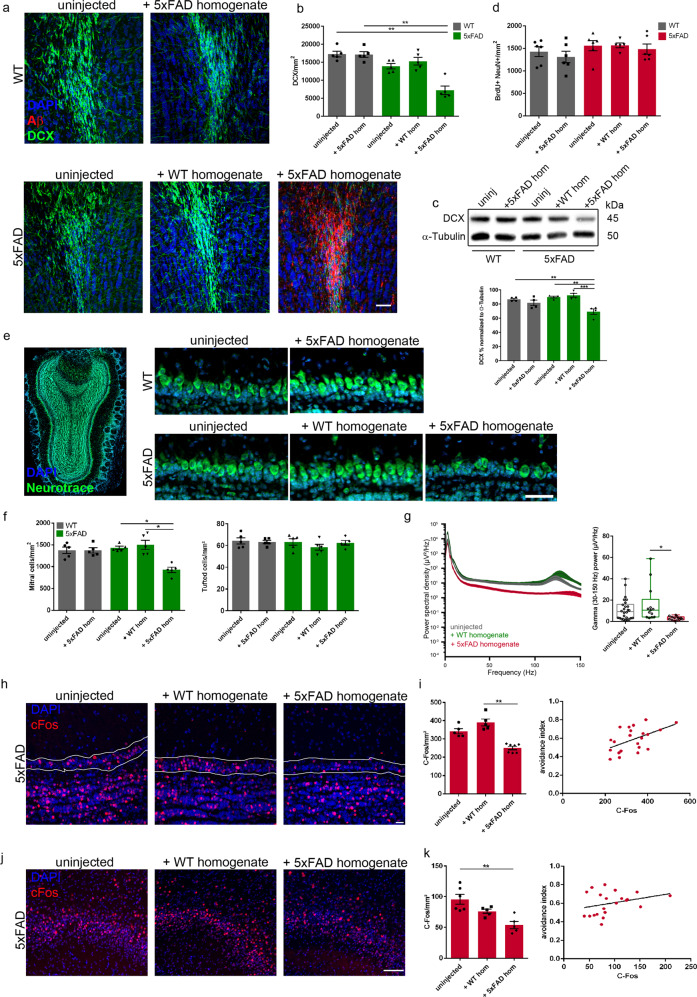
Aβ seeding reduces adult neurogenesis, neuronal function and activity. **a** Fluorescence microscopy of Aβ seeding (6E10, red), DCX (green) and DAPI (blue). Shown are representative images of olfactory bulbs from mice sacrificed at the age of 5 months (12 weeks p.i.). Mice were WT or 5xFAD mice uninjected, injected with WT or 5xFAD brain homogenate. Scale bar represents 50 μm. **b** Quantification of DCX‐positive cells in the olfactory bulb of WT and 5xFAD mice uninjected, injected with WT or 5xFAD homogenate. Each symbol represents data from one mouse (*n* = 5 animals per group were analyzed). Data are presented as mean ± s.e.m. Significant differences were determined by one‐way ANOVA followed by Tukey’s multiple comparison test F(4, 20) = 19.69, *p* = 0.0059; *p* = 0.0094. **c** Immunoblots were conducted on lysates of the olfactory bulb from 5-month-old WT and 5xFAD mice, uninjected or injected with WT or 5xFAD homogenate. The graph represents the relative protein level of DCX normalized to α-Tubulin. Each symbol represents data from one mouse (*n* = 4 animals per group). Data are presented as mean ± s.e.m. Significant differences were determined by one-way ANOVA. F(4, 15) = 10.24, *p* = 0.0048; *p* = 0.0048; *p* = 0.0012, *p* = 0.0004. **d** Quantification of BrdU/NeuN positive cells in the olfactory bulb of WT and 5xFAD. Each symbol represents data from one mouse (WT uninjected, WT + 5xFAD and 5xFAD + 5xFAD *n* = 6, 5xFAD uninjected and 5xFAD + WT *n* = 5). Data are presented as mean ± s.e.m. F(4, 24) = 0.9347. **e** Representative images of Neurotrace staining of 5‐month‐old WT or 5xFAD mice, uninjected or injected with WT or 5xFAD homogenate. Shown is an overview of the olfactory bulb and inserts of the mitral cell layer. Scale bar represents 50 μm. **f** Quantification of Neurotrace positive cells in the mitral cell layer (left) or the external plexiform layer (right) of WT and 5xFAD. Each symbol represents data from one mouse. Data are presented as mean ± s.e.m. Significant differences were determined by one‐way ANOVA followed by Tukey’s multiple comparison test F(4, 20) = 10.44, *p* = 0.034; *p* = 0.0198. *N* = 5 animals per group were analyzed. **g** Power spectral density analysis in the mitral cell layer as a function of frequency (left) and combined for the gamma frequency range (30–150 Hz, right). Each symbol represents data from one penetration (uninjected: 26 electrodes, 3 mice, wt-injected: 13 electrodes, 2 mice, seeded: 13 electrodes, 2 mice). Data are represented as mean ± s.e.m. Significant differences were determined by Kruskal-Wallis test followed by Tukey’s multiple comparison test F(2, 49) = 4.5, *p* = 0.0019. **h** Representative images of cFos (red) staining in the mitral cell layer of 5xFAD mice, uninjected or injected with WT or 5xFAD homogenate. Scale bar represents 20 μm. **i** Quantification of cFos positive cells in the mitral cell layer (left). Data are presented as mean ± s.e.m. Significant differences were determined by one‐way ANOVA followed by Tukey’s multiple comparison test F(2, 14) = 29.27, *p* = 0.0017. Pearson correlation between cFos positive cells in the mitral cell layer and the avoidance index (from Fig. 2) (right). Each symbol represents data from one mouse (5xFAD uninjected and 5xFAD + WT *n* = 5, 5xFAD + 5xFAD *n* = 7), (*r* = 0.5214; *p* = 0.0128). **j** Representative images of cFos (red) staining in the piriform cortex of 5xFAD mice, uninjected or injected with WT or 5xFAD homogenate. Scale bar represents 100 μm. **k** Quantification of cFos positive cells in the piriform cortex (left). Data are presented as mean ± s.e.m. Significant differences were determined by one‐way ANOVA followed by Tukey’s multiple comparison test F(2, 13) = 11.01, *p* = 0.0058. Pearson correlation between cFos positive cells in the piriform cortex and the avoidance index (from Fig. 2) (right). Each symbol represents data from one mouse (5xFAD uninjected *n* = 6, 5xFAD + WT and + 5xFAD *n* = 5), (*r* = 0.298, *p* = 0.01885).

Based on the finding that induced Aβ deposits are primarily located in the subependymal layer and the aco where neuroblasts reach the olfactory bulb to become interneurons, we used DCX, a well-established marker for neurogenesis expressed by immature newborn neurons, and found significantly less DCX positive neurons in seeded 5xFAD mice relative to controls (Fig. 3a, b). Likewise, immunoblotting confirmed lower DCX levels in the olfactory bulb of seeded mice (Fig. 3c). Immunfluorescence of the proliferation marker Ki67 and Sox2 revealed a decreased amount of proliferating neural precursor cells in the SVZ (Supplementary Fig. 3a, b) and ependymal layer (Supplementary Fig. 3c, d). Again, this lower amount of Sox2-expressing neural stem cells was confined to the ependymal layer and coincided with the seeding formation in 5xFAD mice (Supplementary Fig. 3c, d). Interestingly, the number of Calretinin positive granule cells was as well significantly reduced in seeded 5xFAD mice (Supplementary Fig. 3e, f). However, this drop in newborn neuron numbers was not reflected by the proliferative activity since the total number of BrdU positive cells in the whole granule cell layer remained unchanged in all experimental groups (Fig. 3d). Nevertheless, the number of BrdU positive cells in the principal type of projecting neurons, the mitral cells, was substantially decreased in those seeded mice (Supplementary Fig. 4a–c), putting the mitral cell layer center stage. Therefore, we intended to study in detail whether Aβ seeding had an effect on the mitral cell layer visualized by NeuroTrace staining (green fluorescent Nissl stain) and observed indeed a reduction in the number of mitral cells in the seeded mice (Fig. 3e, f left), whereas the number of tufted cells in the external plexiform layer did not change (Fig. 3f right). It is well known that fast gamma oscillations rely on the dendrodendritic interaction between excitatory mitral cells and inhibitory granule cells [44–46]. Thus, to assess whether the OB was functionally impaired in seeded mice, we conducted local field potential recordings from anesthetized mice. Spectral analysis of electrodes located in the mitral cell layer revealed a significant reduction in the power of spontaneous gamma oscillations, suggesting indeed impaired activity of mitral cell-granule cell loops (Fig. 3g). This impairment was observed only in seeded mice suggesting that it is not caused by the injection itself because the control injected mice showed normal gamma oscillation.

In order to further investigate odor-induced neuronal activity, we focused on cFos expression as a reporter for activated neurons [47, 48] in brain regions known to be involved in olfaction. As a first step we determined the number of cFos positive labeled cells in the mitral cell layer. Consistent with our electrophysiological results, we found significantly lower numbers of cFos-positive cells in seeded mice when compared to controls (Fig. 3h, i). This finding raised the question if Aβ seeding would also have an impact on those brain areas, which receive direct projections from the olfactory bulb such as e.g., the piriform cortex, which is involved in olfaction as well although no seeding could be observed in this region. Notably, we obtained similar results when we quantified the number of cFos positive cells in the piriform cortex (Fig. 3j, k). Moreover, the performance in the olfactory avoidance test correlated with the amount of cFos positive cells insofar that with better test performance more cFos labeled cells were present in the brain (Fig. 3i, k). We thus conclude that seed-induced Aβ plaques disturb adult neurogenesis and neuronal activity, further supporting the concept of seeding-evoked diminished olfactory functionality.

### Enriched environment prevents Aβ seeding in the olfactory bulb and reverses olfactory deficits

**Fig. 4:**
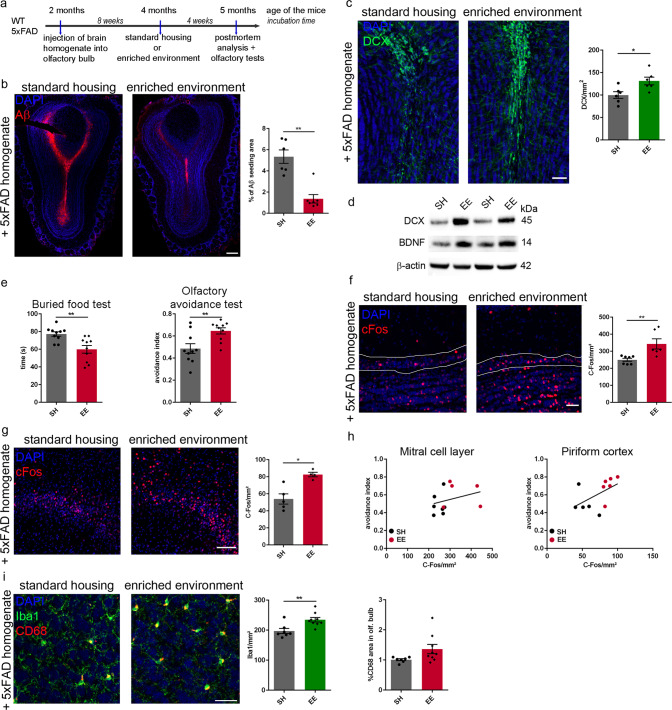
Environmental enrichment diminishes Aβ seeding, stimulates neurogenesis and reverses olfactory deficits in seeded 5xFAD mice. **a** Scheme of the experimental protocol for housing mice in SH or EE. **b** Fluorescence microscopy of Aβ seeding (6E10, red) and DAPI (blue). Shown are representative images from 5xFAD mice injected with 5xFAD homogenate and housed under SH (left) or EE (right) conditions. Scale bar represents 200 μm. The graph represents the quantification of induced Aβ plaque load in olfactory bulb of 5xFAD mice housed in SH or EE. Each symbol represents data from one mouse (SH *n* = 6, EE *n* = 7). Data are presented as mean ± s.e.m. Significant differences were determined by the Mann-Whitney test F(1, 11) = 5.463, *p* = 0.0023. **c** Fluorescence microscopy of DCX (green) and DAPI (blue). Shown are representative images from 5xFAD mice injected with 5xFAD homogenate and housed under SH (left) or EE (right) conditions. Scale bar represents 50 μm. Graph represents the number of DCX-positive cells in the olfactory bulb of 5xFAD mice housed in SH or EE. Each symbol represents data from one mouse (*n* = 6). Data are presented as mean ± s.e.m. Significant differences were determined by unpaired *t*-test F(1, 10) = 2.789, *p* = 0.0191. **d** Representative immunoblots of olfactory bulb lysates from 5-month-old 5xFAD mice injected with 5xFAD brain homogenate and housed under SH or EE conditions. Immunoblot was probed with antibody that recognize DCX and BDNF. β-Actin was used as loading control. **e** Quantification of the buried food (left) and the olfactory avoidance test (right). Data are presented as mean ± s.e.m. Each symbol represents data from one mouse (*n* = 10). Significant differences were determined by unpaired t-test F(1, 18) = 2.983, *p* = 0.003; F(1, 18) = 2.441, *p* = 0.0069. **f** Representative images of cFos (red) staining in the mitral cell layer of 5xFAD mice injected with 5xFAD homogenate and housed in SH or EE. Quantification of cFos positive cells in the mitral cell layer (right). Each symbol represents data from one mouse (SH *n* = 7, EE *n* = 6). Data are presented as mean ± s.e.m. Significant differences were determined by the unpaired *t*-test F(1, 11) = 9.448, *p* = 0.0088. Scale bar represents 20 μm. **g** Representative images of cFos (red) staining in the piriform cortex of 5xFAD mice injected with 5xFAD homogenate and housed in SH or EE. Quantification of cFos positive cells in the piriform cortex (right). Each symbol represents data from one mouse (SH *n* = 5, EE *n* = 4). Data are presented as mean ± s.e.m. Significant differences were determined by the unpaired *t*-test F(1, 7) = 6.392, *p* = 0.0159. Scale bar represents 100 μm. **h** Pearson correlation between cFos positive cells in the mitral cell layer and the avoidance index (*r* = 0.31) (left) and Pearson correlation between cFos positive cells in the piriform cortex and the avoidance index (r = 0.527) (right). Each symbol represents data from one mouse. **i** Fluorescence microscopy of Iba1 (green) and CD68 (red). Shown are representative images from seeded 5xFAD mice. Mice were housed in SH (left) or EE (right). Scale bar represents 20 μm. Quantification of Iba1-positive cells in the olfactory bulb of 5xFAD mice injected with Aβ‐containing brain homogenate (left). Percentage of CD68‐positive area in the olfactory bulb of seeded 5xFAD mice housed in SH or EE (right). Each symbol represents data from one mouse (SH *n* = 7, EE *n* = 9). Data are presented as mean ± s.e.m. Significant differences were determined by the unpaired *t*-test F(1, 14) = 1.029, *p* = 0.0053; F(1,14) = 28.22, *p* = 0.0719.

There is a wide range of studies reporting beneficial effects of an environmental enrichment (EE) and voluntary running on adult hippocampal neurogenesis, Aβ plaque pathology and behavior [22, 49–51]. However, information on the effect of EE on the olfactory bulb are rare and inconsistent [52–54]. In order to determine the effect of EE on Aβ seeding in the olfactory bulb, we injected 5xFAD mice with transgenic homogenate and exposed them 8 weeks p.i. for another 4 weeks either to EE or SH (Fig. 4a). Remarkably, the seeding capacity of mice housed in an EE was dramatically reduced (Fig. 4b) whereas the number of DCX positive cells on the contrary was significantly increased in the EE group (Fig. 4c). Immunoblotting confirmed higher DCX levels in the olfactory bulb of seeded 5xFAD mice housed in EE (Fig. 4d). Furthermore, elevated levels of BDNF in the EE group imply a stimulation of adult neurogenesis via neurotrophic factors in this context (Fig. 4d). Strikingly, olfactory deficits induced by Aβ seeding could be rescued by housing the mice in an EE as detected by the buried food and the avoidance test (Fig. 4e). Moreover, by quantifying cFos positive cells in the mitral cell layer and the piriform cortex, it was clearly evident that the group of mice housed in EE had significantly more activated neurons compared to their counterparts housed in SH (Fig. 4f, g). Concomitantly, this result correlated with the performance in the olfactory avoidance test, indicating that more cFos positive cells resulted in better performance in the olfactory avoidance test (Fig. 4h). Consistent with an earlier study where we reported that exposure to EE reduces Aβ seeding in the hippocampus by activating phagocytic microglia [22], we found significantly more Iba1 positive cells in the granule cell layer of the olfactory bulb in mice exposed to EE compared to mice housed in SH (Fig. 4i). Likewise, the percentage of CD68 abundance was enhanced in the EE group but did not reach significance (Fig. 4i). Inflammatory cytokine analysis by qRT-PCR showed increased levels of *TNfα* and *Ccl2* in seeded mice housed in EE, which was also confirmed by western blot analysis (Supplementary Fig. 5a, b). Together, these data support the idea that EE can modulate early phases and signs of AD pathology including olfactory deficits.

## Discussion

Several recent studies have implicated olfactory loss in neurodegenerative diseases such as Parkinson’s and Alzheimer’s disease [4–6], but this relation has remained ambiguous. Our study presents converging evidence for detrimental effects of seed-induced Aβ deposits on olfaction. Aβ seeding, which resembles early stages of Aβ plaque formation exerted a direct negative influence on neuronal development, neuronal activity and function. Moreover, by modulating Aβ seeding via enriched housing conditions these neuronal malfunctions were restored finally leading again to improved olfaction. Our findings thus provide insight into an Aβ driven mechanism for the loss of olfaction in a mouse model of AD. The seeding model of AD pathology in mice offers a unique tool for studying Aβ plaque formation in vivo at its very early stage and within a defined period of time. The biggest advantage for using seeding in the olfactory bulb is that we can study the direct effect of Aβ on olfaction without aging.

Only a few studies have addressed olfactory behavior in AD mouse models in the past, however with different outcomes. While one study reported intact odor discrimination but deficits in olfactory memory [55], others uncovered no apparent dysfunction in odor detection but rather detected pronounced odor habituation/dishabituation changes [56, 57]. Hence, we set out to investigate the formation of Aβ deposits in respect to olfactory performance in an in vivo seeding model. This model has the advantage to display Aβ plaque formation at its very early stage within a defined time period and that therefore the age of the newborn plaques is easily determinable due to the predictability of the model [58]. We first established Aβ seeding in the olfactory bulb in two different APP transgenic mouse models. Importantly, in both, the extent and the progression of Aβ seeding was similar with a considerable amount of Aβ seeding already 12 weeks p.i. in 5xFAD mice. Although at this age, seed-induced Aβ pathology was restricted to the injection site (subependymal layer and aco), neuritic plaques also become evident in the subependymal layer and granule cell layer across the bulb in uninjected control 5xFAD mice at 8 months of age [59]. Further studies need to investigate spreading to other brain regions.

We had previously shown that Aβ seeding in the hippocampus induced neuronal cell death leading to memory deficits [22]. In agreement with this finding, we indeed discovered problems in olfaction and olfactory discrimination in seeded mice that reached a certain level of induced Aβ, providing evidence that Aβ might be the driver of olfactory deficits. Because Aβ seeding occurred predominantly in the subependymal layer and the aco of the olfactory bulb where adult born neurons from the SVZ arrive, mature to interneurons and integrate into the olfactory network [60–62], we hypothesized that adult neurogenesis is impaired in those mice as well. In fact, the number of DCX positive cells was dramatically decreased most likely due to fewer cells that are generated in the SVZ although the question still remains whether more dying cells on their way towards the olfactory bulb might account for this. Nevertheless, the overall number of BrdU/NeuN positive cells was similar, indicating that most of the cells reaching the olfactory bulb also integrate in the granule cell layer. Since Aβ seeding affected bulbar neurogenesis as well as some other neuronal populations in the olfactory bulb such as the mitral cells, we decided to analyze bulbar physiology with a focus on the mitral cell layer. Our finding of reduced spontaneous gamma oscillations is in line with the fact that gamma oscillations become altered when projection neurons or their circuitry are damaged thus lowering olfactory abilities [63]. Moreover, our result that shows the strong impairment of the mitral cell layer even without hardly any noticeable proximity to the seeding pattern points towards a disrupted network/connectivity between the different types of neurons involved in olfaction. It is important to note that the control injected animals did not show any deficits, which makes it clear that the injection alone did not cause the deficits. Furthermore, reduced cFos expression in mitral cells and piriform cortex correlated with the performance in the olfactory tests providing a mechanistic link. These data fit to our previous observations based on Aβ seeding in the hippocampus [22] and support the notion that seed-induced Aβ deposits may be a source of toxicity with functional relevance.

Enriched environment, physical activity and voluntary running have shown great promise for many different conditions [49, 50] and individual laboratories have reported ameliorated neuropathological AD phenotypes [22, 51, 64–67], but the potential beneficial effects for the olfactory bulb and olfaction in AD mouse models has been so far completely overlooked. Intriguingly, both our data on adult neurogenesis and the activity measure of mitral cells and neurons in the piriform cortex indicate a reversal to normal levels in mice exposed to EE. Reminiscent of our previous study performed in the hippocampus, we hypothesize that Aβ seeding was reduced possibly due to activated microglia under EE housing, finally leading to improved olfactory behavior. Collectively, our study elucidates a mechanism by which Aβ seeding initiates a pathological process that possibly culminates in olfactory dysfunction and highlights EE as a promising approach to ameliorate these early pathological effects with the potential to even restore olfactory function.

## Supplementary information

Supplemental Information

## Data Availability

The data that support the findings of this study are available from the corresponding author upon reasonable request.
