# Supplementary material for: Seed-induced Aβ deposition alters neuronal function and impairs olfaction in a mouse model of Alzheimer’s disease
Source: Mol Psychiatry. 2022 Jul 22;27(10):4274–84. doi: 10.1038/s41380-022-01686-5 (PMC9718674; doi:10.1038/s41380-022-01686-5)
Supplement: Supplementary file 1 — Supplemental Information [file 41380_2022_1686_MOESM1_ESM.docx]

**Supplemental Material**

**Seed-induced A**β **deposition alters neuronal function and impairs olfaction in a mouse model of Alzheimer’s disease**

Stephanie Ziegler-Waldkirch, Marina Friesen, Desirée Loreth, Jonas-Frederic Sauer, Solveig Kemna, Alexandra Hilse, Daniel Erny, Christina Helm, Paolo d´Errico, Marco Prinz, Marlene Bartos & Melanie Meyer-Luehmann


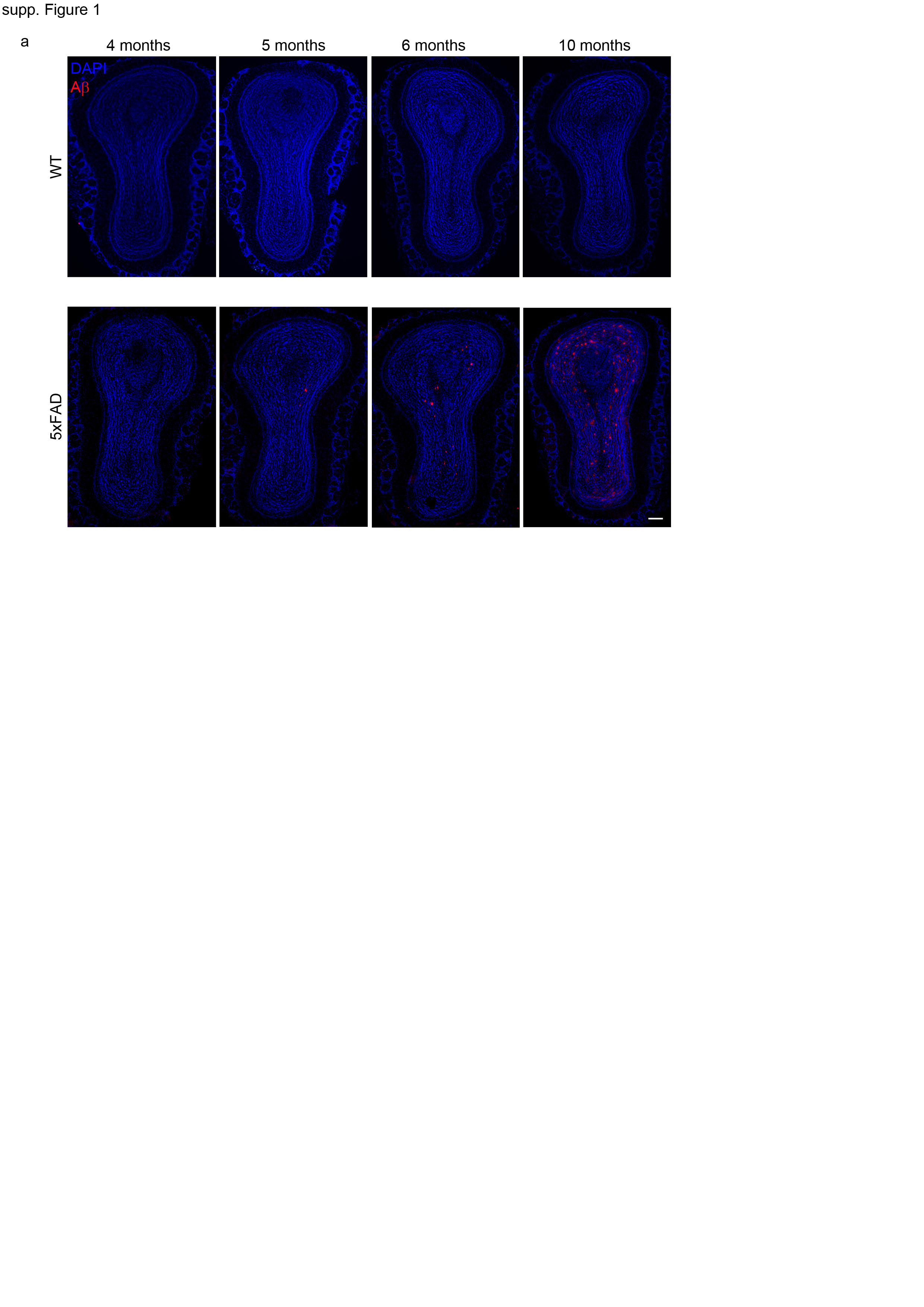


**SUPPLEMENTARY FIGURE 1. Characterization of Aβ plaque load in the olfactory bulb of 5xFAD mice.**

Fluorescence microscopy of Aβ plaques (6E10, red) and DAPI (blue). Shown are representative images of a 4, 5, 6 and 10‐month‐old uninjected male 5xFAD mice. First Aβ plaques can be seen in the granule cell layer at 6 months of age. Scale bar represents 200 μm.


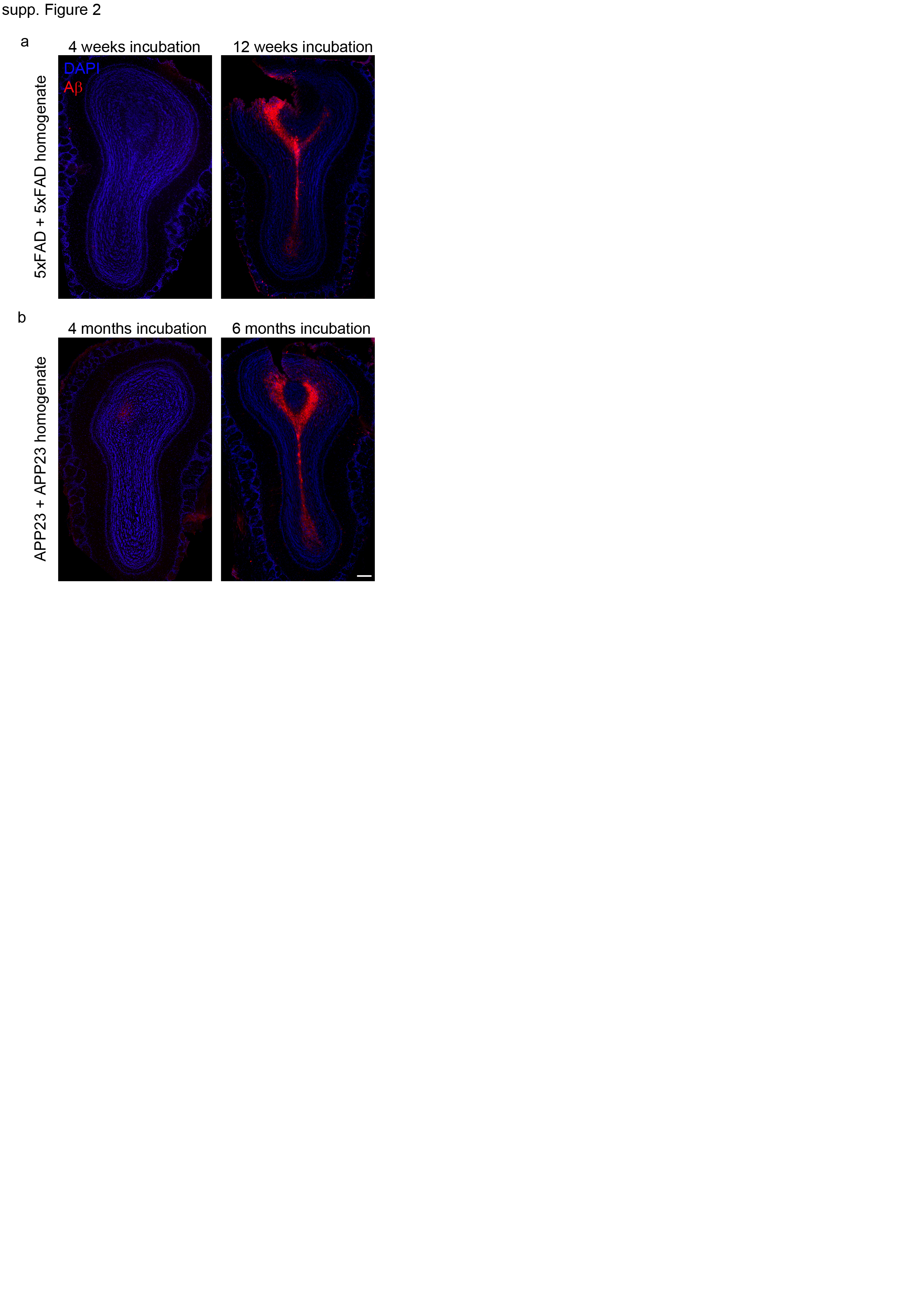


**SUPPLEMENTARY FIGURE 2. Aβ seeding in 5xFAD and APP23 mice.**

(a) Fluorescence microscopy of Aβ seeding (6E10, red) and DAPI (blue) in the olfactory bulb of 5xFAD mice injected with 5xFAD homogenate, 4 and 12 weeks p.i. Scale bar represents 200 μm.

(b) Fluorescence microscopy of Aβ seeding (6E10, red) and DAPI (blue) in the olfactory bulb of APP23 mice injected with APP23 homogenate, 4 and 6 months p.i. Aβ seeding is located in the anterior commissure of the olfactory bulb. Scale bar represents 200 μm.


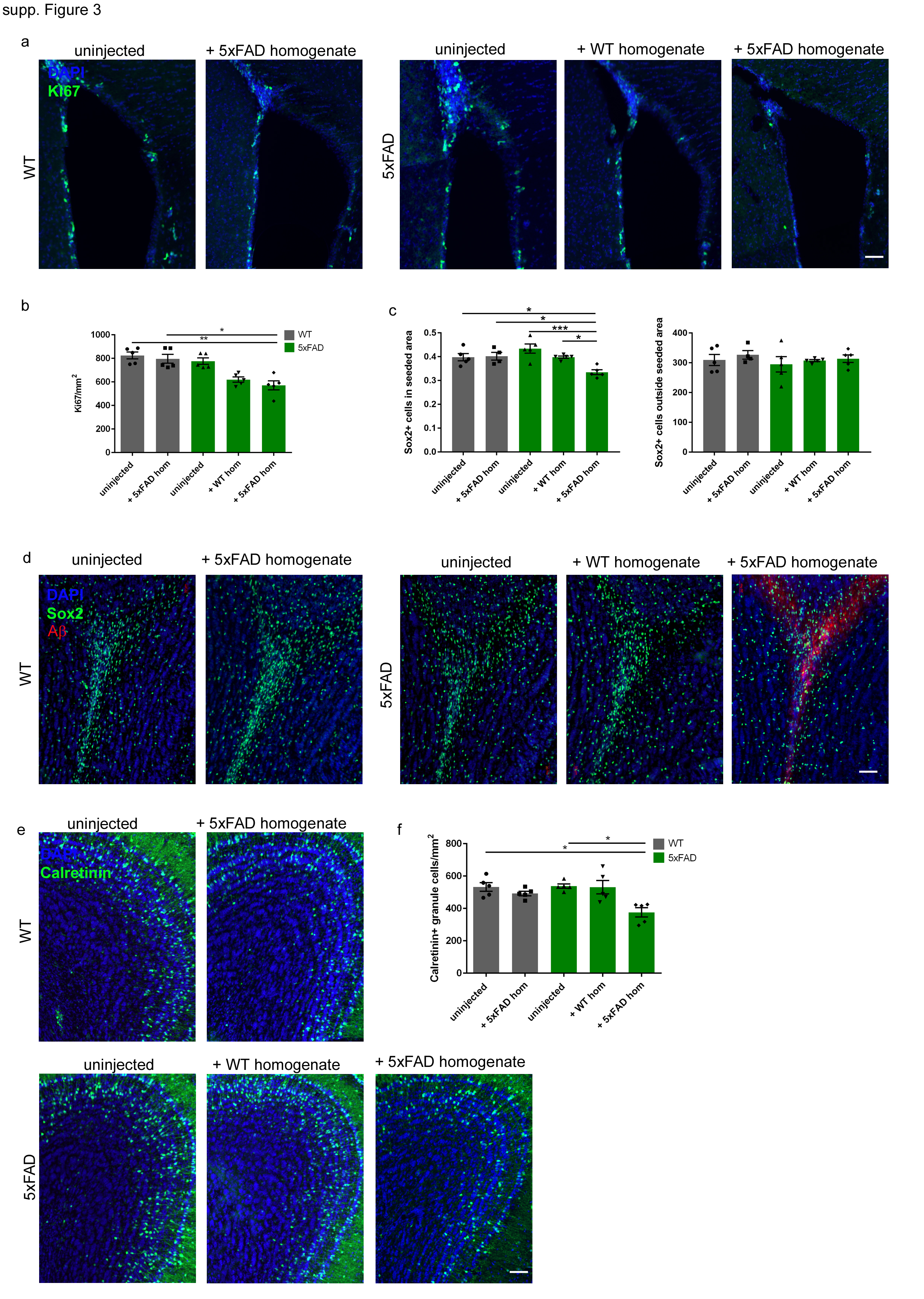


**SUPPLEMENTARY FIGURE 3. Aβ seeding alters neuronal population in the olfactory bulb.**

(a) Fluorescence microscopy of Ki67 (green) and DAPI (blue) in the subventricular zone (SVZ). Shown are representative images from WT and 5xFAD mice uninjected, injected with WT or 5xFAD homogenate. Scale bar represents 50 μm.

(b) Graph represents the number of Ki67-positive cells in the SVZ. Each symbol represents data from one mouse (n=5 in each group). Data are presented as mean ± s.e.m. Significant differences were determined by one‐way ANOVA followed by Tukey's multiple comparison test (F(4, 20)=13.07, p=0.0228, p=0.008).

(c) Quantification of the Sox2 positive cells in the olfactory bulb in the seeding area (left graph) and outside the seeding area (right graph) (n=4 for WT injected with 5xFAD homogenate, all other groups n=5). Data are presented as mean ± s.e.m. Significant differences were determined by one‐way ANOVA followed by Tukey's multiple comparison test (F(4,19)=6.85, F(4,19) =0.4248, p=0.0306, p=0.0299, p=0.0005, p=0.0306).

(d) Fluorescence microscopy of Sox2 (green), Aβ (red) and DAPI (blue) in the granule cell layer of the olfactory bulb. Shown are representative images from WT and 5xFAD mice uninjected, injected with WT or 5xFAD homogenate. Scale bar represents 50 μm.

(e) Fluorescence microscopy of Calretinin (green) and DAPI (blue) in the granule cell layer of the olfactory bulb. Shown are representative images from WT and 5xFAD mice uninjected, injected with WT or 5xFAD homogenate. Scale bar represents 50 μm.

(f) Quantification of Calretinin positive cells in the granule cell layer of the olfactory bulb (n=5 in each group). Data are presented as mean± s.e.m. Significant differences were determined by one‐way ANOVA followed by Tukey's multiple comparison test (F(4,20)=6.534, p=0.0303, p=0.0171).


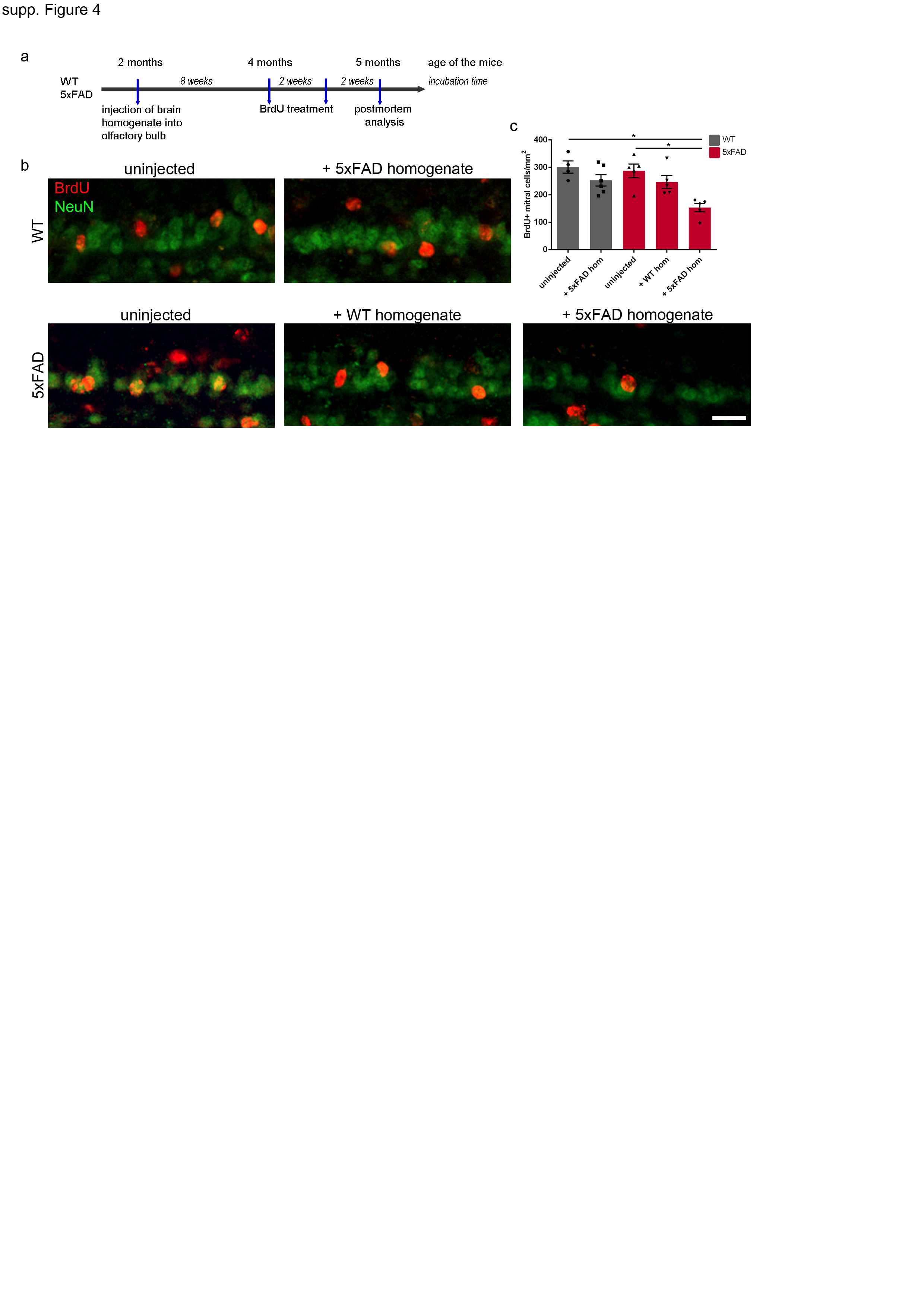


**SUPPLEMENTARY FIGURE 4. Seed**‐**induced Aβ deposits reduce proliferation/survival of newborn neurons in the olfactory bulb.**

(a) Scheme of the experimental protocol for BrdU treatment.

(b) Fluorescence microscopy of BrdU (red) and NeuN (green). Shown are representative images of the mitral cell layer from WT mice and 5xFAD mice uninjected or injected with WT or 5xFAD brain homogenate. Scale bar represents 50 μm.

(c) Quantification of BrdU/NeuN‐positive cells in the mitral cell layer of WT (n=4) and 5xFAD (n=5) mice uninjected or injected with WT or 5xFAD homogenate (n=5). Each symbol represents data from one mouse. Data are presented as mean ± s.e.m. Significant differences were determined by one‐way ANOVA followed by Tukey's multiple comparison test (F(4,20)=6.361, p=0.0119; p=0.0303).


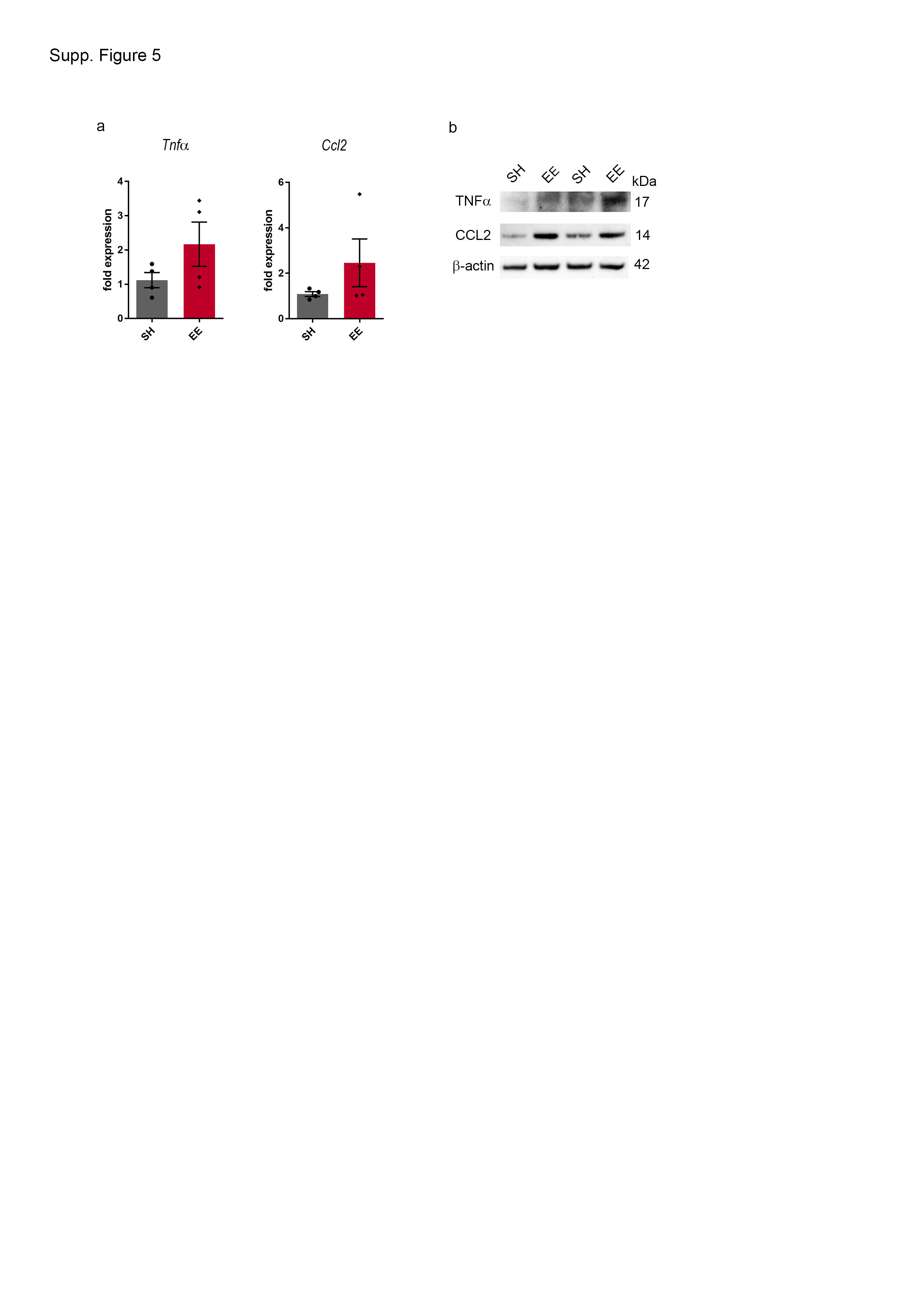


**SUPPLEMENTARY FIGURE 5. Housing under EE conditions leads to increased cytokine levels.**

(a) qRT-PCR of *Cc*l2 and *Tnfα* from olfactory bulb lysate samples from 5xFAD mice injected with 5xFAD brain homogenate housed under SH or EE conditions. Data are expressed as a ratio of the mRNA expression compared with endogenous *Actb.* Data are presented as mean ± s.e.m.

(b) Representative immunoblots of olfactory bulb lysates from 5-month-old 5xFAD mice injected with 5xFAD brain homogenate and housed under SH or EE conditions. Immunoblots were probed with antibodies that recognize CCL2 and TNFα. β-Actin was used as loading control.
